# Prediction of the potential distribution of the predatory mite *Neoseiulus californicus* (McGregor) in China under current and future climate scenarios

**DOI:** 10.1038/s41598-022-15308-1

**Published:** 2022-07-12

**Authors:** Lin Chen, Chunxian Jiang, Xueyan Zhang, Cancan Song, Rulin Wang, Xian Wang, Qing Li

**Affiliations:** 1 College of Agronomy, Sichuan Agricultural University, Chengdu, 611130 Sichuan China; 2 Sichuan Provincial Rural Economic Information Centre, Chengdu, 610072 Sichuan China

**Keywords:** Climate-change ecology, Ecological modelling, Zoology

## Abstract

*Neoseiulus californicus* is a predatory mite with a wide global distribution that can effectively control a variety of pest mites. In this study, MaxEnt was used to analyse the potential distribution of *N. californicus* in China and the BCC-CSM2-MR model was used to predict changes in the suitable areas for the mite from 2021 to 2100 under the scenarios of SSP126, SSP245 and SSP585. The results showed that (1) the average of area under curve value of the model was over 0.95, which demonstrated an excellent model accuracy. (2) Annual mean temperature (Bio1), precipitation of coldest quarter (Bio19), and precipitation of driest quarter (Bio17) were the main climatic variables that affected and controlled the potential distribution of *N. californicus*, with suitable ranges of 6.97–23.27 °C, 71.36–3924.8 mm, and 41.94–585.08 mm, respectively. (3) The suitable areas for *N. californicus* were mainly distributed in the southern half of China, with a total suitable area of 226.22 × 10^4^ km^2^ in current. Under the future climate scenario, compared with the current scenario, lowly and moderately suitable areas of *N. californicus* increased, while highly suitable areas decreased. Therefore, it may be necessary to cultivate high-temperature resistant strains of *N. californicus* to adapt to future environmental changes.

## Introduction

*Neoseiulus californicus* (McGregor) (Acari: Phytoseiidae) is a very effective predator of spider mites and was first described on lemons trees in California, USA, in 1954^1^. *N. californicus* is widely distributed in Argentina, Chile, the United States, South Africa, Japan, Southern Europe, the Mediterranean coast and other subtropical and tropical areas, mainly inhabiting citrus, grapes, strawberries, avocados, maize, cassava, vegetables and other plants^2–4^. Controlling pest mites with *N. californicus* is a biological control method with many advantages. First, using *N. californicus* to control harmful mites does not cause environmental pollution, can effectively control the mites to a certain extent, and has many applications in orchard and vegetable fields^5,6^. Vidrih et al.^7^ reported that satisfactory results could be achieved in suppressing *Tetranychus urticae* Koch on a hop plantation by repeated use of the predatory mite *N. californicus*. Second, *N. californicus* can prey on a variety of pest mites, including *Tetranychus cinnabarinus* (Boisduval)^8^, *Panonychus citri* (McGregor)^9^, *T. urticae*^2^, *Polyphagotarsonemus latus* (Banks)^10^, and *Panonychus ulmi* (Koch)^11^, worldwide. Finally, *N. californicus* can be used in combination with agents to improve management and reduce the use of chemical pesticides. Sato et al.^12^ found that it was feasible to control *T. urticae* in strawberry fields using selective acaricides and *N. californicus*. Although abamectin, acephate, fenpropathrin, iprodione, mineral oil and tebuconazole were shown to be slightly harmful to *N. californicus*, a combination of the two can be used for integrated control^13^.

Species Distribution Models (SDMs) are designed to correlate the distribution data of species with the corresponding environmental variables (climate, soil, vegetation, elevation, host, etc.) of their distribution sites, analyse the relationship between the geographical distribution of the species and the environmental variables, and build models^14–16^. Currently, the MaxEnt model^17^, GARP model^18^, Bioclim model^19^ and domain model are commonly used. MaxEnt models the geographical distribution of species according to the niche theory proposed by Jaynes^20–22^ and has been used widely since its launch in 2004 to model such distributions^23–25^. The salient features of MaxEnt are that it requires only presence data and can use both continuous and categorical data^26^. In addition, it is simple to use and can give reliable and stable output even with small datasets^27,28^. In addition, many studies have indicated that the MaxEnt model has a better simulation effect among various species distribution models. Elith et al.^29^ compared the simulation performance of various niche models, and the results showed that MaxEnt had the highest prediction accuracy among 16 models. Currently, MaxEnt can be used for the analysis of species habitat requirements^30^, impact of future climate change on species distribution^31^, species invasion monitoring^32–34^ and natural analysis of protected areas^35^.

Since *N. californicus* is a predatory mite that can control a variety of pest mites, the prediction and simulation of the potential distribution area of *N. californicus* is of great significance for its application in the future. In this paper, the distribution record points of *N. californicus* were determined by consulting the literature and the Global Biodiversity Information Facility (GBIF) and Centre Agriculture Bioscience International (CABI) websites. The future distribution of *N. californicus* in China was predicted with the MaxEnt model under the SSP126, SSP245 and SSP585 scenarios. This study had three objectives: (1) to evaluate the main environmental variables afecting the distribution of *N. californicus*, (2) to explore the distribution of *N. californicus* under current and future climatic scenarios, and to provide a theoretical basis for how to use *N. californicus* to control pests in different periods and places in the future, (3) determine the changes in the suitable distribution area under the future scenario model, and provide biological protection recommendations on how to respond to climate change.

## Results

### Accuracy evaluation of the MaxEnt model

The accuracy test of the MaxEnt model is shown in Table 1.The results showed that the AUC value of the training dataset was 0.9762 and that of the test dataset was 0.9706 and value of the TSS was 0.7839 under the current climate conditions. In future climate scenarios, the AUC values of the SSP126, SSP245 and SSP585 scenarios of the training data were 0.9741–0.9766, 0.9748–0.9756, and 0.9748–0.9762, respectively, and those of the test data were 0.9670–0.9694, 0.9682–0.9696, and 0.9679–0.9708, respectively. The AUC values were higher than 0.9 and the ROC curve extended upwards to the left, indicating that the simulation results of all the constructed models were considered to be good and could be used for subsequent analysis.

**Table 1:** AUC values of MaxEnt model for *N. californicus* under climate change scenarios.

Scenarios	Period	Training data ± SE	Test data ± SE
Current (1970–2000)	–	0.9762 ± 0.0004	0.9706 ± 0.0041
SSP126	2030s	0.9747 ± 0.0004	0.9689 ± 0.0044
	2050s	0.9750 ± 0.0005	0.9683 ± 0.0056
	2070s	0.9766 ± 0.0004	0.9694 ± 0.0048
	2090s	0.9741 ± 0.0004	0.9670 ± 0.0056
SSP245	2030s	0.9754 ± 0.0005	0.9682 ± 0.0052
	2050s	0.9756 ± 0.0003	0.9686 ± 0.0035
	2070s	0.9748 ± 0.0005	0.9690 ± 0.0059
	2090s	0.9756 ± 0.0004	0.9696 ± 0.0042
SSP585	2030s	0.9756 ± 0.0003	0.9696 ± 0.0043
	2050s	0.9762 ± 0.0004	0.9708 ± 0.0033
	2070s	0.9755 ± 0.0005	0.9679 ± 0.0063
	2090s	0.9748 ± 0.0002	0.9693 ± 0.0028

### Key climatic variables affecting the occurrence of *N. californicus*

**Figure 1:**
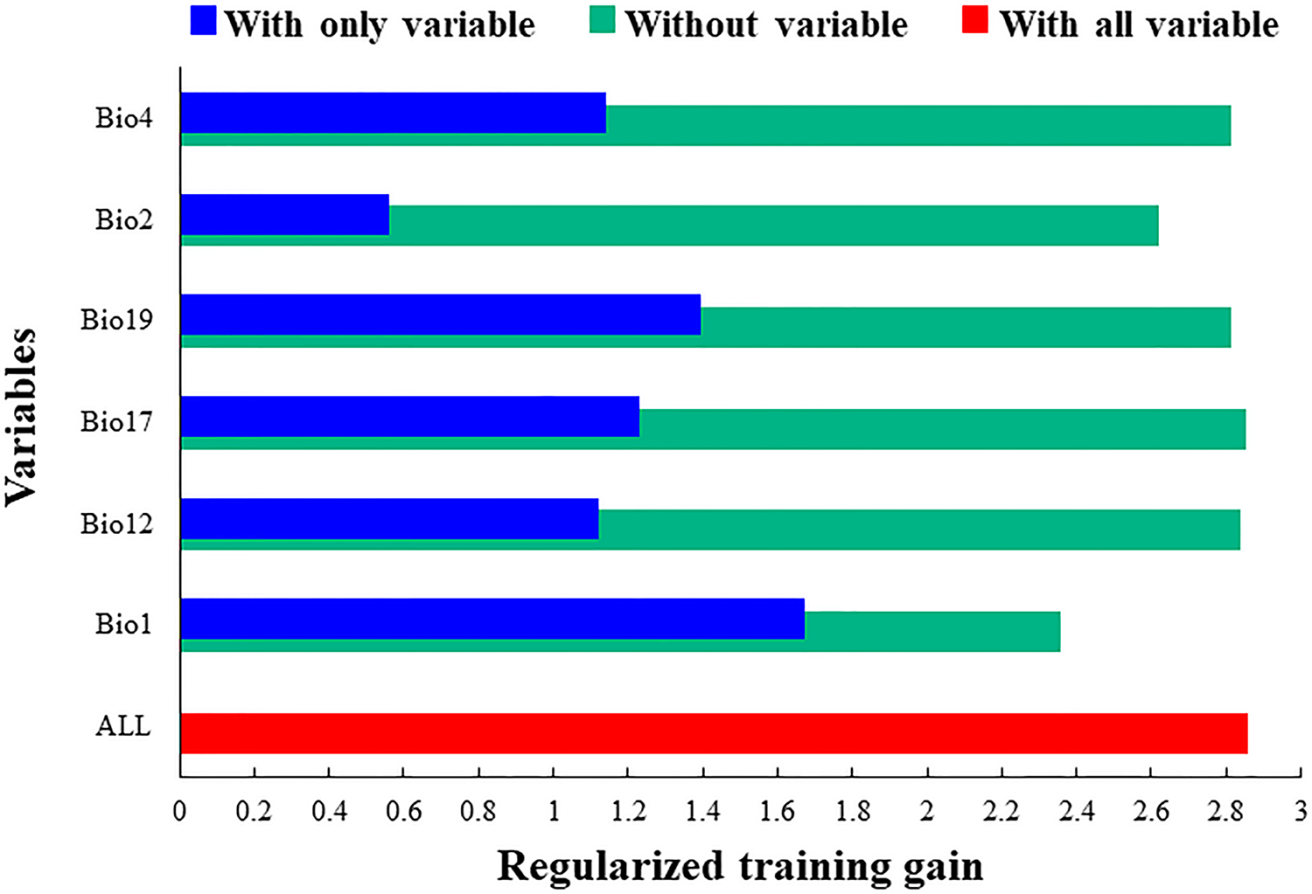
Important analysis of environmental variables based on Jackknife tests.

**Figure 2:**
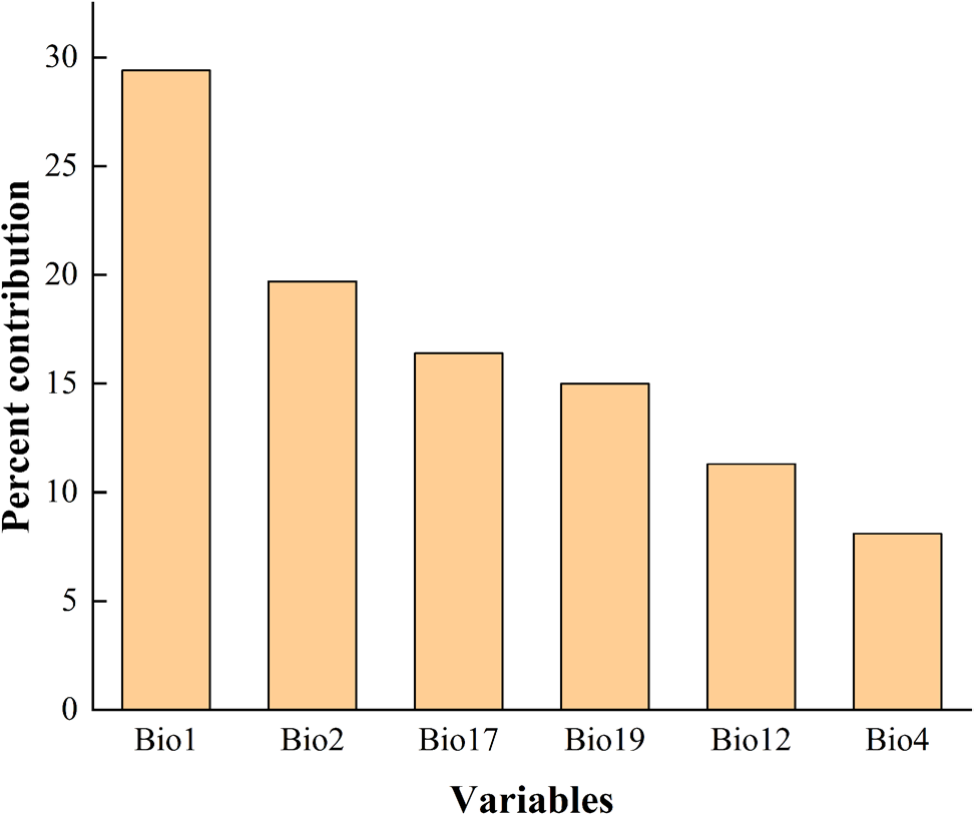
Analysis of the importance of environmental variables.

The importance of the six main variables that had a great influence on the distribution of *N. californicus* was compared by the Jackknife method, and the results are shown in Fig. 1. The longer the blue band was, the more important the variable was to the distribution of the species. Combined with the contribution rate of these environmental variables to the species (Fig. 2), the three most important environmental variables for *N. californicus* were Bio1 (29.4%), Bio19 (15%), and Bio17 (16.4%).

**Figure 3:**
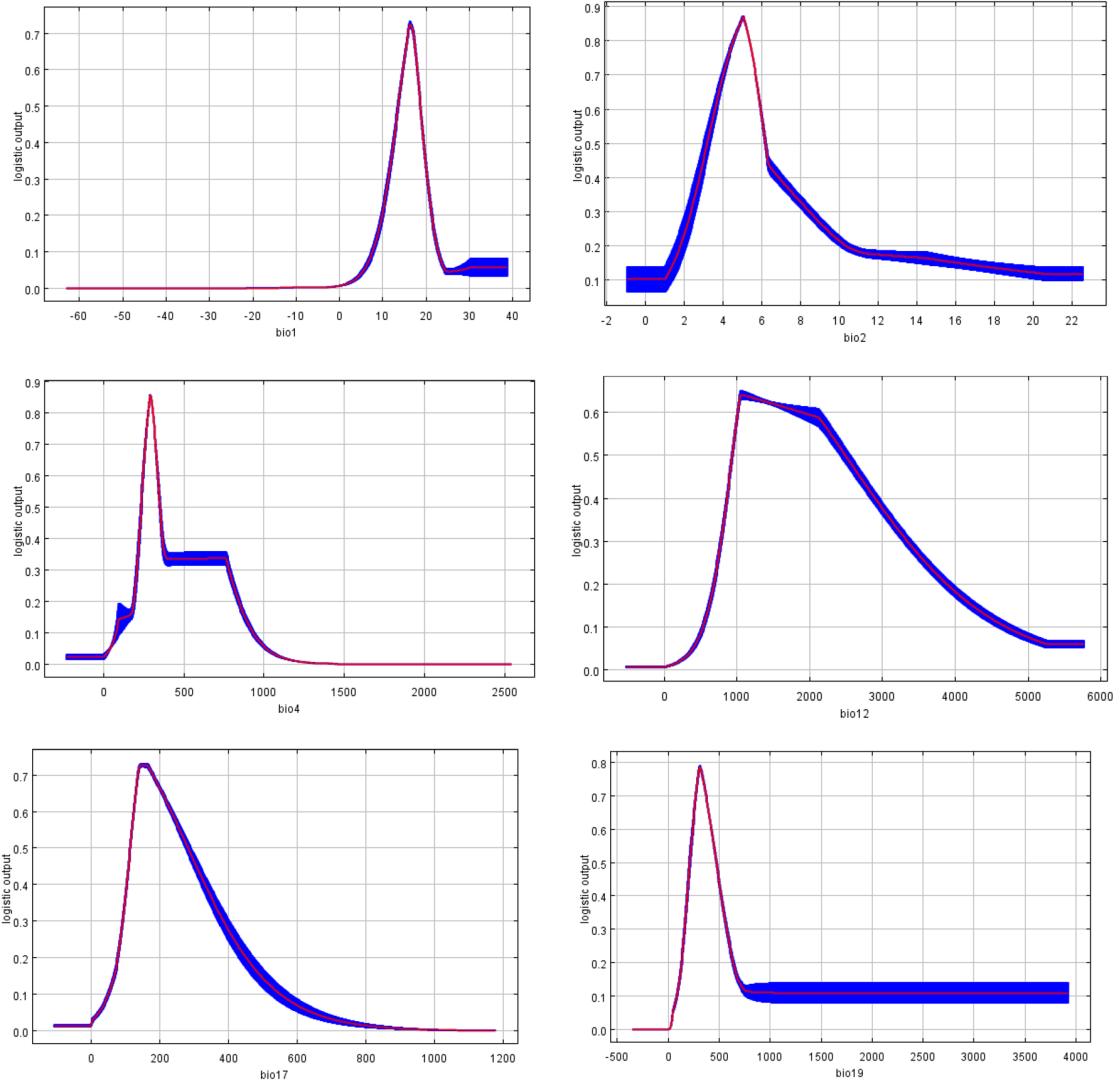
Response curves between the probability of presence and environmental variables.

The response curves output by MaxEnt in this study reflected the suitability thresholds for the main environmental variables that affected the distribution of *N. californicus*. The distribution probability of *N. californicus* increased with the increase in environmental variables within a certain range and decreased with the increase in variables after reaching a certain peak value. The results showed that the appropriate range ranges of Bio1, Bio19, Bio17, Bio4, Bio12, and Bio2 were 6.97–23.27 °C, 71.36–3924.8 mm, 41.94–585.08 mm, 65.63–958.57 °C, 488.74–4975.51 mm and -0.96–22.57 °C, respectively (Fig. 3).

### Potential distribution areas of *N. californicus* in China under current scenarios

**Figure 4:**
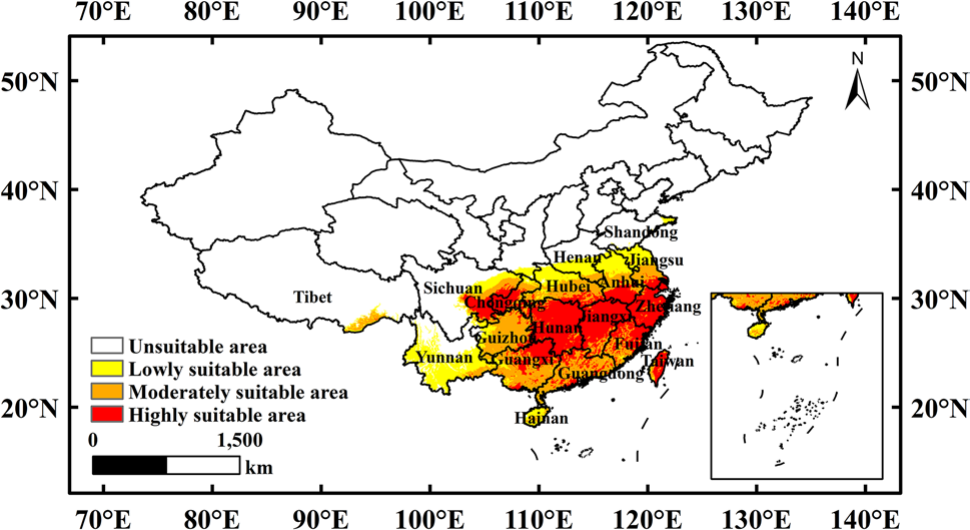
Potential suitable distribution of *N. californicus* in China based on MaxEnt under the current scenarios. Created in ESRI ArcMap 10.8.1 (https://support.esri.com/en/Products/Desktop/arcgis-desktop/arcmap).

As shown in Fig. 4 and Table 2, the highly suitable areas for *N. californicus* in China were mainly distributed in Hunan, Jiangxi, Zhejiang, Guangxi, Fujian, Sichuan, Guangdong, Chongqing, Anhui, Hubei, Taiwan, Guizhou, Jiangsu and other regions, with an area of 90.92 × 10^4^ km^2^, accounting for 9.48% of China's land area. Among these provinces, Hunan (19.5 × 10^4^ km^2^) and Jiangxi (15.01 × 10^4^ km^2^) had the largest distribution area. The moderately suitable areas were mainly distributed in Guangxi, Guizhou, Guangdong, Hubei, Sichuan, Fujian, Anhui, Jiangsu and other provinces, with a total area of 77.09 × 10^4^ km^2^, accounting for 8.04% of China's land area. Among these provinces, the largest distribution areas were Guangxi (13.23 × 10^4^ km^2^), Guizhou (12.67 × 10^4^ km^2^) and Guangdong (12.03 × 10^4^ km^2^). The unsuitable area was located in northern Sichuan, Shaanxi, Henan and Shandong, with a total area of 733.03 × 10^4^ km^2^, accounting for 76.42% of China's land area.

**Table 2:** Predicted areas for *N. californicus* under current climatic conditions.

Province	Unsuitable area		Lowly suitable area		Moderately suitable area		Highly suitable area	
	Area (× 10^4^ km^2^)	Proportion	Area (× 10^4^ km^2^)	Proportion	Area (× 10^4^ km^2^)	Proportion	Area (× 10^4^ km^2^)	Proportion
Beijing	1.64	0.22	0.00	0.00	0.00	0.00	0.00	0.00
Tianjin	1.19	0.16	0.00	0.00	0.00	0.00	0.00	0.00
Hebei	18.72	2.55	0.00	0.00	0.00	0.00	0.00	0.00
Shanxi	15.67	2.14	0.00	0.00	0.00	0.00	0.00	0.00
Inner Mongolia	119.61	16.32	0.00	0.00	0.00	0.00	0.00	0.00
Liaoning	14.81	2.02	0.00	0.01	0.00	0.00	0.00	0.00
Jilin	19.02	2.60	0.00	0.00	0.00	0.00	0.00	0.00
Heilongjiang	43.97	6.00	0.00	0.00	0.00	0.00	0.00	0.00
Shanhai	0.00	0.00	0.00	0.00	0.00	0.00	0.63	0.70
Jiangsu	0.37	0.05	4.22	7.26	4.14	5.37	1.50	1.65
Zhejiang	0.00	0.00	0.00	0.00	0.53	0.68	9.82	10.80
Anhui	0.09	0.01	5.49	9.43	4.32	5.61	4.06	4.46
Fujian	0.00	0.00	0.20	0.35	4.34	5.64	7.74	8.51
Jiangxi	0.00	0.00	0.00	0.00	1.70	2.21	15.01	16.50
Shandong	14.69	2.00	1.12	1.93	0.01	0.01	0.00	0.00
Henan	10.00	1.36	5.51	9.47	1.16	1.51	0.00	0.00
Hubei	0.56	0.08	5.91	10.16	8.73	11.33	3.37	3.71
Hunan	0.00	0.00	0.00	0.01	1.68	2.18	19.50	21.45
Guangdong	0.00	0.00	0.04	0.08	12.03	15.61	5.35	5.88
Guangxi	0.00	0.00	1.60	2.75	13.23	17.16	8.92	9.81
Hainan	0.08	0.01	2.15	3.70	0.84	1.09	0.03	0.03
Chongqing	0.09	0.01	1.05	1.81	2.30	2.98	4.79	5.27
Sichuan	34.46	4.70	4.83	8.30	4.47	5.80	5.41	5.95
Guizhou	0.85	0.12	2.11	3.62	12.67	16.44	1.99	2.19
Yunnan	16.55	2.26	20.57	35.35	2.15	2.79	0.13	0.14
Tibet	115.76	15.79	1.75	3.01	1.86	2.42	0.03	0.03
Shaanxi	19.11	2.61	1.46	2.50	0.00	0.00	0.00	0.00
Gansu	45.49	6.21	0.00	0.00	0.00	0.00	0.00	0.00
Qinghai	69.66	9.50	0.00	0.00	0.00	0.00	0.00	0.00
Ningxia	6.64	0.91	0.00	0.00	0.00	0.00	0.00	0.00
Xinjiang	164.00	22.37	0.00	0.00	0.00	0.00	0.00	0.00
Hong Kong	0.00	0.00	0.00	0.00	0.00	0.00	0.11	0.12
Taiwan	0.00	0.00	0.17	0.29	0.91	1.19	2.54	2.79
Total	733.03		58.21		77.09		90.92	

### Potential distribution areas of *N. californicus* in China under future climatic scenarios

**Figure 5:**
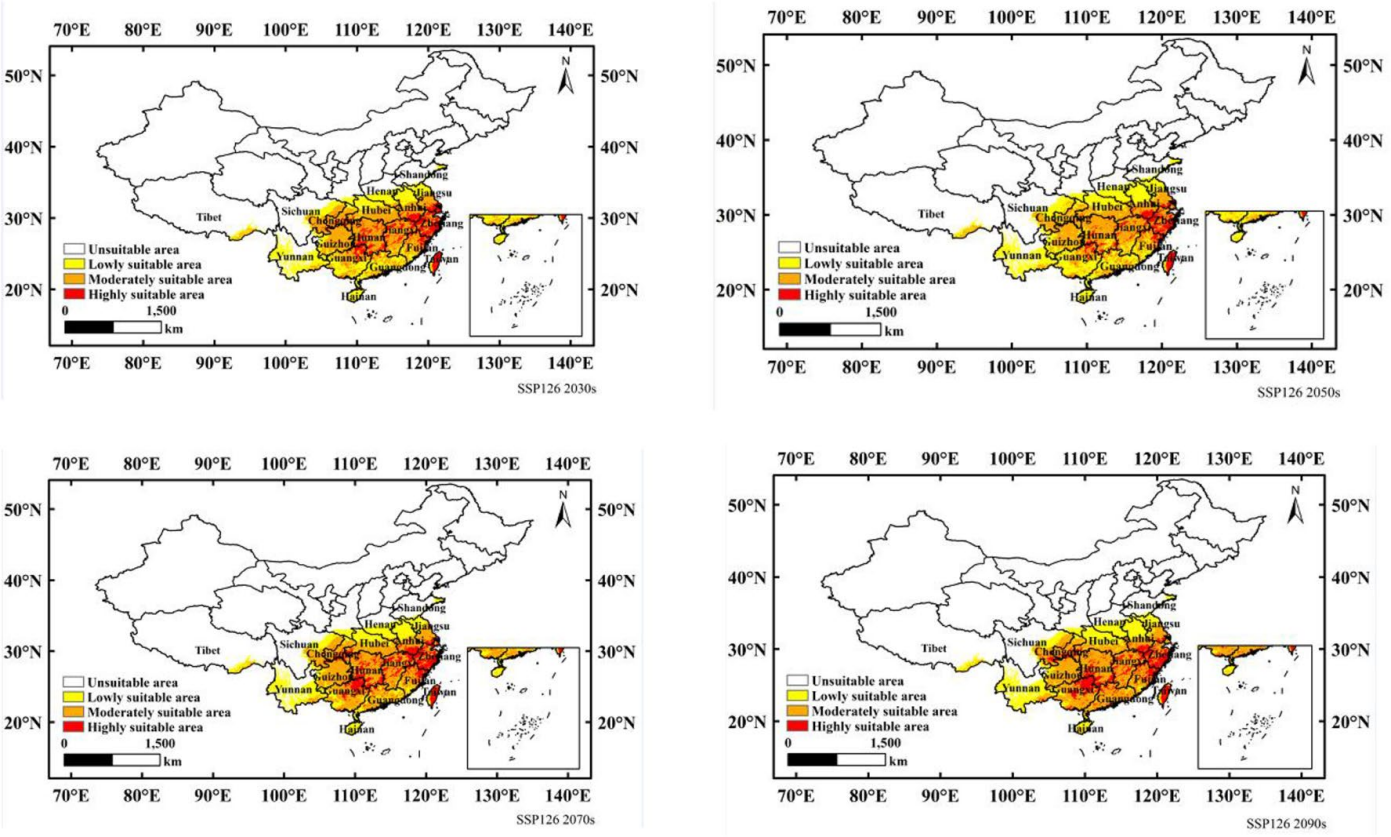
Potential suitable distribution of *N. californicus* in China based on MaxEnt under scenario of SSP126. Created in ESRI ArcMap 10.8.1 (https://support.esri.com/en/Products/Desktop/arcgis-desktop/arcmap).

Figure 5 shows the suitable distribution of *N. californicus* in China in the 2030s, 2050s, 2070s and 2090s under the SSP126 scenario. As shown in Fig. 5 and Table 3, compared with the current scenario, the lowly and moderately suitable areas increased significantly, while the highly suitable areas decreased significantly. The low suitable area will increase from a current area of 58.21 × 10^4^ km^2^ to 75.41 × 10^4^ km^2^ (2030s), 93.11 × 10^4^ km^2^ (2050s), 69.14 × 10^4^ km^2^ (2070s) and 67.45 × 10^4^ km^2^ (2090s) in the future. The moderately suitable area was projected to increase from a current area of 77.09 × 10^4^ km^2^ to 101.17 × 10^4^ km^2^ (2030s), 94.85 × 10^4^ km^2^ (2050s), 103.93 × 10^4^ km^2^ (2070s) and 107.05 × 10^4^ km^2^ (2090s). The highly suitable area was projected to decrease from a current area of 90.92 × 10^4^ km^2^ to 29.79 × 10^4^ km^2^ (2030s), 19.76 × 10^4^ km^2^ (2050s), 35.98 × 10^4^ km^2^ (2070s) and 35.98 × 10^4^ km^2^ (2090s).

**Table 3:** Predicted areas of suitable areas for *N. californicus* under climate changes cenarios.

Scenario	Period	Lowly suitable area		Moderately suitable area		Highly suitable area	
		Predicted area (× 10^4^ km^2^)	Proportion of current Predicted area (%)	Predicted area (× 10^4^ km^2^)	Proportion of current Predicted area (%)	Predicted area (× 10^4^ km^2^)	Proportion of current Predicted area (%)
	Current	58.21	**-**	77.09	-	90.92	**-**
SSP126	2030s	75.41	129.56	101.17	131.24	29.78	32.76
	2050s	93.11	159.96	94.85	123.04	19.76	21.73
	2070s	69.14	118.78	103.93	134.82	35.96	39.56
	2090s	67.45	115.87	107.05	138.87	35.98	39.58
SSP245	2030s	65.58	112.67	88.60	114.93	50.13	55.14
	2050s	66.62	114.46	110.18	142.92	29.43	32.37
	2070s	105.01	180.40	79.03	102.52	16.18	17.79
	2090s	108.05	185.62	88.73	115.10	12.49	13.74
SSP585	2030s	62.78	107.85	92.49	119.98	55.67	61.23
	2050s	98.20	168.71	92.62	120.15	20.52	22.57
	2070s	103.28	177.42	89.03	115.49	14.90	16.39
	2090s	148.50	255.12	36.69	47.59	5.57	6.12

**Figure 6:**
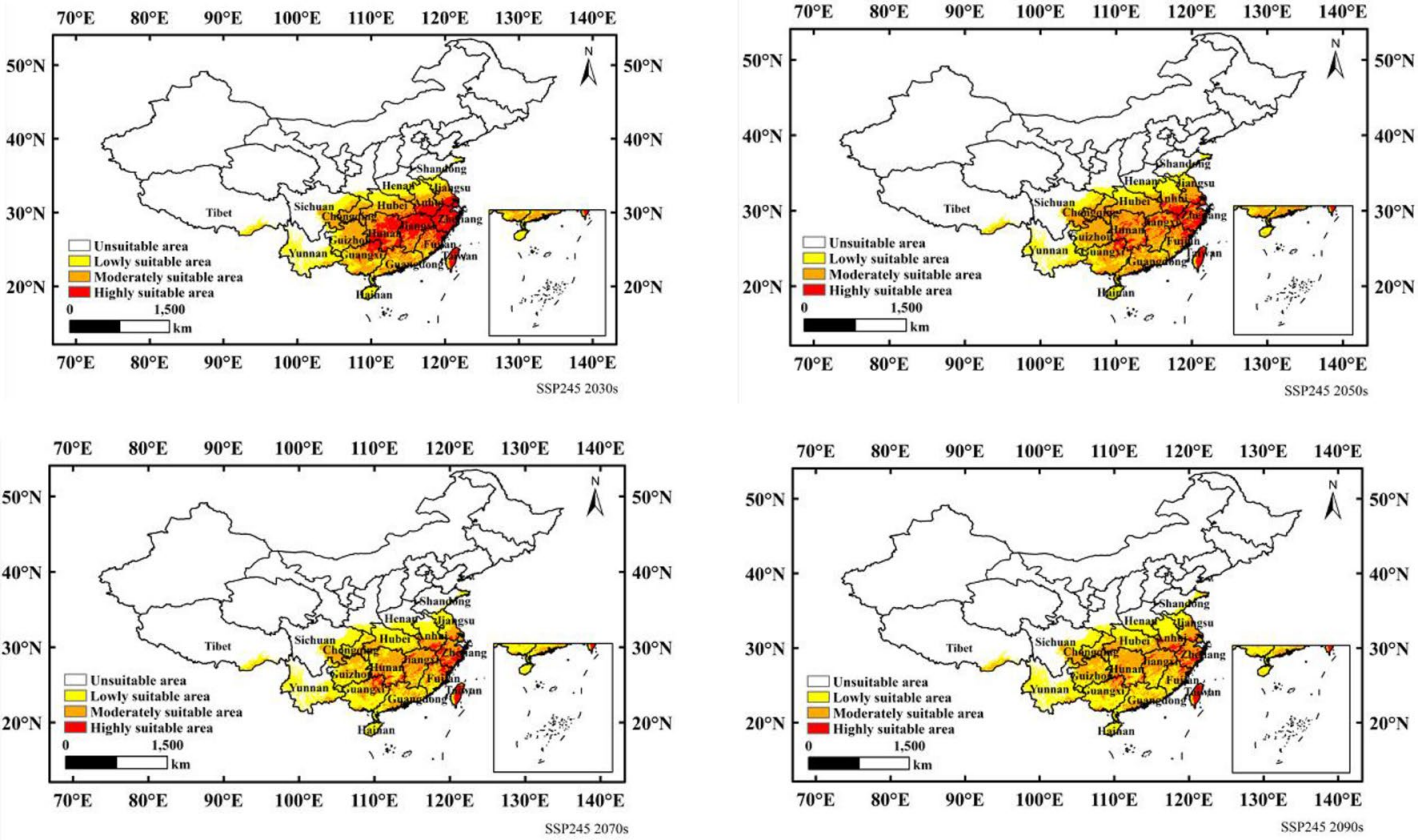
Potential suitable distribution of *N. californicus* in China based on MaxEnt under scenario of SSP245. Created in ESRI ArcMap 10.8.1 (https://support.esri.com/en/Products/Desktop/arcgis-desktop/arcmap).

Figure 6 shows the suitable distribution of *N. californicus* in China in the 2030s, 2050s, 2070s and 2090s under the SSP245 scenario. As shown in Fig. 6 and Table 3, compared with the current scenario, the areas of lowly and moderately suitable increased significantly, while the areas of highly suitable decreased significantly. The low suitable area was projected to increase from a current area of 58.21 × 10^4^ km^2^ to 65.58 × 10^4^ km^2^ (2030s), 66.62 × 10^4^ km^2^ (2050s), 105.01 × 10^4^ km^2^ (2070s) and 108.05 × 10^4^ km^2^ (2090s) in the future. The moderately suitable area was projected to increase from a current area of 77.09 × 10^4^ km^2^ to 88.60 × 10^4^ km^2^ (2030s), 110.18 × 10^4^ km^2^ (2050s), 79.03 × 10^4^ km^2^ (2070s) and 88.73 × 10^4^ km^2^ (2090s). The highly suitable area was projected to decrease from a current area of 90.92 × 10^4^ km^2^ to 50.13 × 10^4^ km^2^ (2030s), 29.43 × 10^4^ km^2^ (2050s), 16.18 × 10^4^ km^2^ (2070s) and 12.49 × 10^4^ km^2^ (2090s).

**Figure 7:**
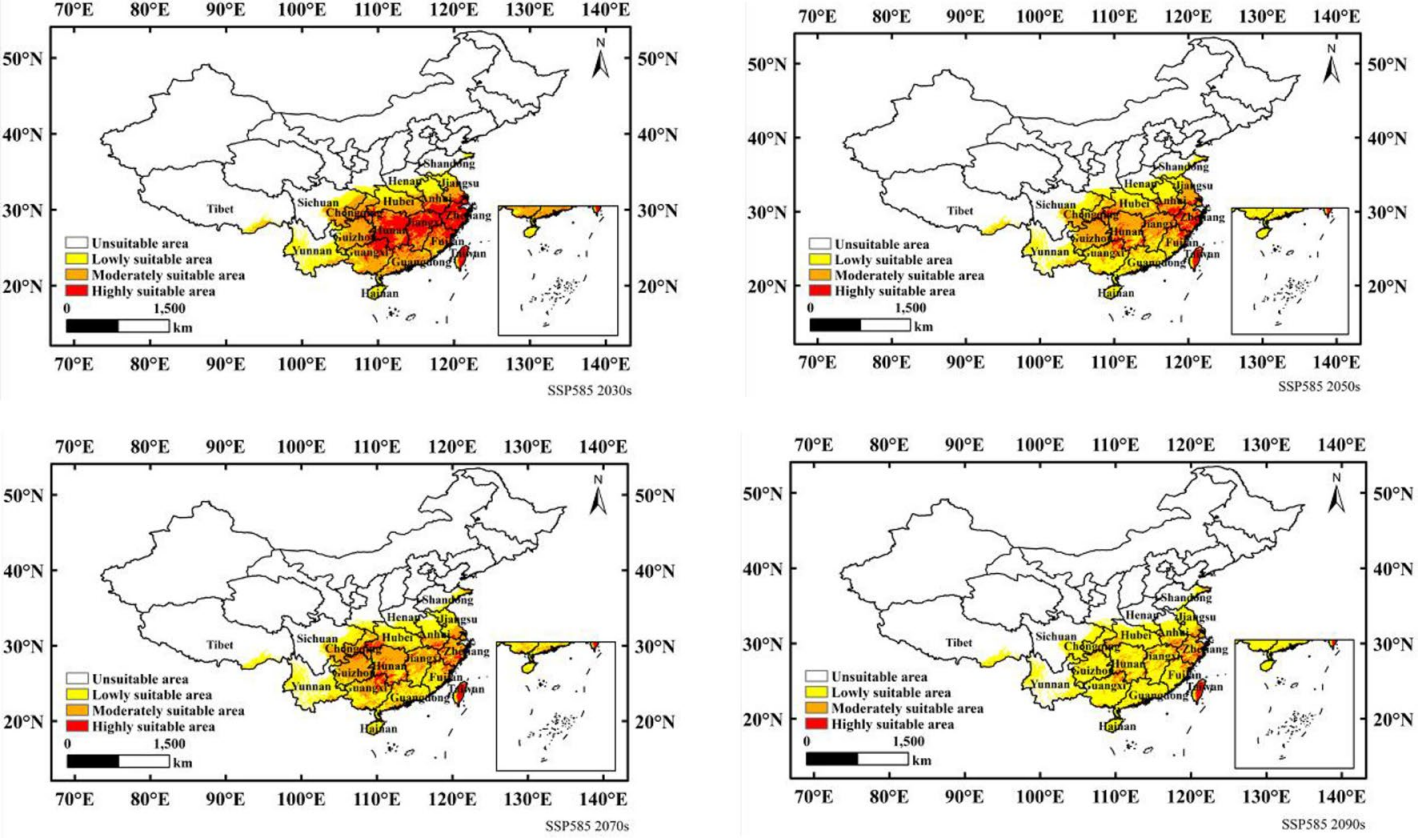
Potential suitable distribution of *N. californicus* in China based on MaxEnt under scenario of SSP585. Created in ESRI ArcMap 10.8.1 (https://support.esri.com/en/Products/Desktop/arcgis-desktop/arcmap).

Figure 7 shows the suitable distribution of *N. californicus* in China in the 2030s, 2050s, 2070s and 2090s under the SSP585 scenario. As shown in Fig. 7 and Table 3, compared with the current scenario, the lowly and moderately suitable areas increased significantly, while the highly suitable area decreased significantly. The low suitable area was projected to increase from a current area of 58.21 × 10^4^ km^2^ to 62.78 × 10^4^ km^2^ (2030s), 98.20 × 10^4^ km^2^ (2050s), 103.28 × 10^4^ km^2^ (2070s) and 148.50 × 10^4^ km^2^ (2090s) in the future. The moderately suitable area was projected to increase from a current area of 77.09 × 10^4^ km^2^ to 92.49 × 10^4^ km^2^ (2030s), 92.62 × 10^4^ km^2^ (2050s), 89.03 × 10^4^ km^2^ (2070s) and reduced to 36.69 × 10^4^ km^2^ (2090s). The highly suitable area was projected to decrease from a current area of 90.92 × 10^4^ km^2^ to 55.67 × 10^4^ km^2^ (2030s), 20.52 × 10^4^ km^2^ (2050s), 14.90 × 10^4^ km^2^ (2070s) and 5.57 × 10^4^ km^2^ (2090s).

### Centroid migration trajectory

In the SSP126 scenario, the species centroids from the current position were 15.64 km (2030s) in the northeast, 0 km (2050s), 8.74 km (2070s) in the west, and 13.47 km (2090s) in the southwest. In the SSP245 scenario, the species centroids from the current position were 23.26 km (2030s) in the northeast, 10.25 km (2050s) in the north, 22.28 km (2070s) in the southwest, and 18.09 km (2090s) in the northwest. In the SSP585 scenario, the species centroids from the current position were 10.71 km (2030s) in the northeast, 34.39 km (2050s) in the northeast, 28.30 km (2070s) in the southwest, and 30.75 km (2090s) in the southeast (Table 4).

**Table 4:** Shift distance and direction of mean centre of different suitable areas of *N. californicus*.

Scenario	Period	Centroid Coordinates		Direction	Displacement (km)
		Longitude (°E)	Latitude (°N)		
	Current	111.4000	27.6669	–	–
SSP126	2030s	111.5224	27.7574	Northeast	15.64
	2050s	111.5224	27.7574	–	0.00
	2070s	111.4319	27.7574	West	8.74
	2090s	111.3413	27.6669	Southwest	13.47
SSP245	2030s	111.6163	27.7574	Northeast	23.26
	2050s	111.6130	27.8479	North	10.25
	2070s	111.5224	27.6669	Southwest	22.28
	2090s	111.3742	27.7646	Northwest	18.09
SSP585	2030s	111.4319	27.7574	Northeast	10.71
	2050s	111.6579	27.9925	Northeast	34.39
	2070s	111.4643	27.8045	Southwest	28.30
	2090s	111.7567	27.6964	Southeast	30.75

## Discussion

*N. californicus*, as a predatory mite, is widely distributed worldwide and can effectively control a variety of pest mites in orchards and vegetable fields. In agriculture, using predatory mites to control agricultural pest mites is an important measure of biological control that has a positive effect on the development of green agriculture. The climate is the largest factor affecting species distribution, and climate change has a great impact on biological diversity and species distribution ranges^36,37^. Since the twentieth century, global climate change has been mainly characterized by warming. The sixth assessment report of the IPCC Working Group I indicated that since 1850–1900, the global average surface temperature has risen by approximately 1 °C and pointed out that from the perspective of the average temperature change over the next 20 years, the global temperature rise is expected to reach or exceed 1.5 ℃^38^. Consequently, the potential distribution of *N. californicus* may change greatly, so the response of the potential distribution of *N. californicus* to future climate change and the shifts and adaptations in its distribution under future climate change scenarios were studied by MaxEnt to facilitate future sustainable development and utilization of *N. californicus*.

In this study, MaxEnt was used to construct a distribution model of *N. californicus* to determine the distribution of this mite in China under current climate conditions and to predict its distribution under different scenarios in the future. As a commonly used model for predicting species distribution, MaxEnt has a wide range of applications and still has good prediction results when species distribution points are small, their numbers are uncertain, or their correlations with environmental variables are unknown^39^. Tognelli et al.^40^ found that MaxEnt had the highest accuracy of all tested models, especially for species sampled from relatively few sites, when determining the distribution of Patagonian insects by artificial neural networks, BIOCLIM, classification and regression trees, DOMAIN, generalized additive models, GARP, generalized linear models, and the MaxEnt model. Pangga et al.^41^ used the MaxEnt model to accurately predict the species distribution of *Aspidiotus rigidus* Reyne, whose main environmental variables were annual temperature variation and seasonality. In addition, before using MaxEnt to predict the distribution, we used the ENMeval package in R to optimize the model and selected a model combination with delta AICc equal to 0 because this tuning exercise can result in a model with a balanced goodness of fit^42^. Ultimately, the AUC values of the distribution data simulated by MaxEnt were all greater than 0.95, so our model was considered robust and sufficient to explain the distribution of *N. californicus*.

Species distribution is highly susceptible to the influence of the environment, and the environment directly or indirectly affects the physiological and ecological functions of species, thereby limiting their distribution^43–45^. In this study, we combined the species distribution points of *N. californicus* and 19 environmental variables to simulate the current suitable distribution of the species with MaxEnt. Combining Jackknife tests and the contribution rate of selected environmental variables with the species locations, Bio1, Bio19 and Bio17 were found to be the main environmental variables affecting the suitable distribution of *N. californicus*. As the most important factor of species distribution, temperature limits the species distribution by affecting its effective accumulation and temperature at the developmental stage. Zhang et al.^46^ reported that *N. californicus* can reproduce normally at 15–35 °C, with the highest net proliferation rate at 25 °C, and its generation growth cycle shortens with increasing temperature. Wang et al.^31^ analysed the current suitable distribution of *N. californicus* and found that Bio19 had an important impact on the distribution of *N. californicus* and that environmental variables related to precipitation in April (prec4), precipitation in June (prec6), precipitation in October (prec10), and precipitation in December (prec12) had important effects on the distribution of *N. californicus*. Because mites are small in size, they are affected by factors such as wind and rain in addition to temperature, and rainfall can negatively affect them by drowning them or knocking them into the soil^47^. The scouring of heavy rain and torrential rain has been reported to have a significant inhibitory effect on *T. cinnabarinus*^48^.

Our results indicated that the total suitable area for *N. californicus* was 226.22 × 10^4^ km^2^. Of this area, the proportion of lowly suitable area accounted for 25.73%, moderately suitable area accounted for 34.08%, and highly suitable area accounted for 40.19%. In general, the boundaries of suitable and unsuitable areas for *N. californicus* were generally consistent with the findings of Wang et al.^31^ In addition, there were also some differences in the locations and areas of different suitable areas. The reason for these differences may be that we obtained 118 points for our distribution prediction after removing redundant points, while Wang et al. obtained 65 points. We performed a model optimization for the parameters in R before running the MaxEnt fitness simulations^31^.

As the global climate warms, the structure and function of terrestrial ecosystems may be significantly altered, resulting in significant changes in the extent and distribution of biological habitats^49^. The latest CMIP6 model shows that the world will be significantly warmed in the future. In the future SSP126, SSP245, and SSP585 scenarios, the temperature is predicted to increase by 1.3–2.9 °C, 2.1–4.3 °C, and 3.8–7.4 °C, respectively^50^. Therefore, based on the current distribution of *N. californicus*, we predicted the potential redistribution of *N. californicus* in response to climate change under these three climate scenarios in the twenty-first century. Our results showed that the size and distribution of the areas of *N. californicus* were different under the three future climate scenarios, but all showed basically the same trends. Compared with the current distribution, the distribution areas of the lowly and moderately of *N. californicus* showed an upwards trend (except in the 2090s under the SSP585 scenario), and the highly suitable area showed a downwards trend. Combined with existing research reports, in the temperature range of 30–35 °C, the mortality of female adult mites significantly increased, indicating that high temperatures above 30 °C have an adverse effect on the development, survival rate and reproduction of *N. californicus*^46^. At 37.5 °C, the females of *N. californicus* can lay eggs, but the eggs cannot hatch; at 40 °C, the females of *N. californicus* cannot lay eggs^51^. This may be one reason why the area of highly suitable area for *N. californicus* was projected to decrease in the future due to climate change. In addition, due to climate change, the overall migration trend of *N. californicus* was to the west (SSP126), northwest (SSP245), and northeast (SSP585).

Climate change and the frequent occurrence of extreme weather will restrict the continuous control of phytoseiid mites in agricultural ecosystems and high-temperature will often interfere with the biological control of tetranychus mites using phytoseius mites. Yuan et al.^52^ found that high-temperature exposure had significant effects on the egg hatching rate, survival rate and development duration of *N. californicus*, but had little effect on the pre-oviposition and survival rate of the adults.Thus, to adapt to the future climate and continue to effectively and continuously control pests and mites in a high-temperature environment, high-temperature resistant strains of *N. californicus* may need to be cultivated in the future as Zhang et al.^53^ selected the high-temperature resistant strain of *Neoseiulus barkeri*.

## Methods

### Software and map sources

MaxEnt software (version 3.4.1)^54^ was downloaded from the Museum of Natural History website (https://biodiversityinformatics.amnh.org/open_source/maxent/); Java software was downloaded from its official website (https://www.oracle.com/java/); R (version 4.1.2)^55^ and RStudio software were downloaded from their official website (https://www.r-project.org/, https://www.rstudio.com/); ArcGIS software (version 10.8.1) was downloaded from the ESRI website (https://support.esri.com/en/Products/Desktop/arcgis-desktop/arcmap); and the base map was provided by the National Meteorological Information Centre of China.

### Occurrence record of *N. californicus*

**Figure 8:**
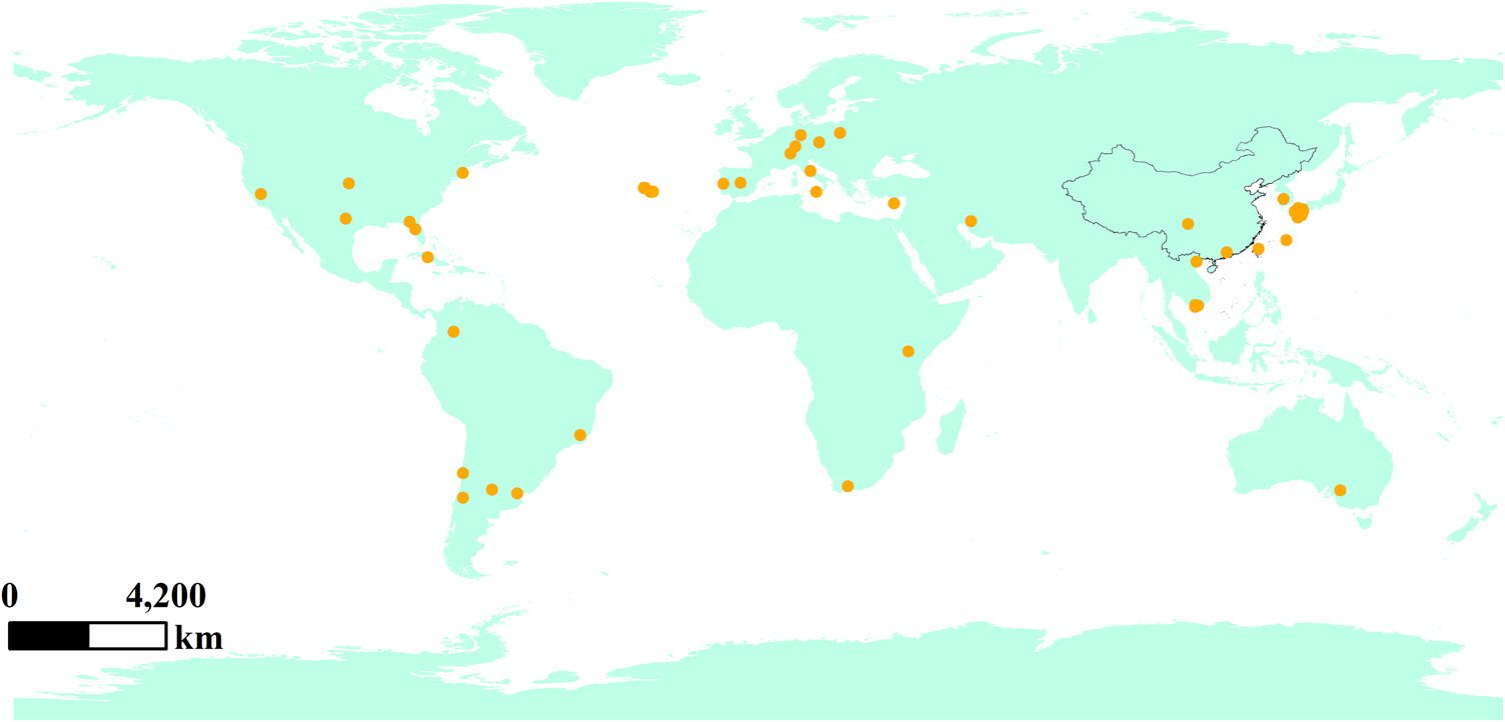
Global distribution of *N. californicus*. Created in ESRI ArcMap 10.8.1 (https://support.esri.com/en/Products/Desktop/arcgis-desktop/arcmap).

The species distribution data of *N. californicus* were downloaded from the GBIF (https://www.gbif.org/) and CABI (https://www.plantwise.org/knowledgebank/) websites and combined with relevant literature on the occurrence of *N. californicus*^4,56–66^*.* The latitude and longitude recorded in the literature for *N. californicus* were determined using Google Earth. Through the above procedure, a total of 118 distribution data points was obtained (Fig. 8).

### Climatic variables related to *N. californicus*

**Figure 9:**
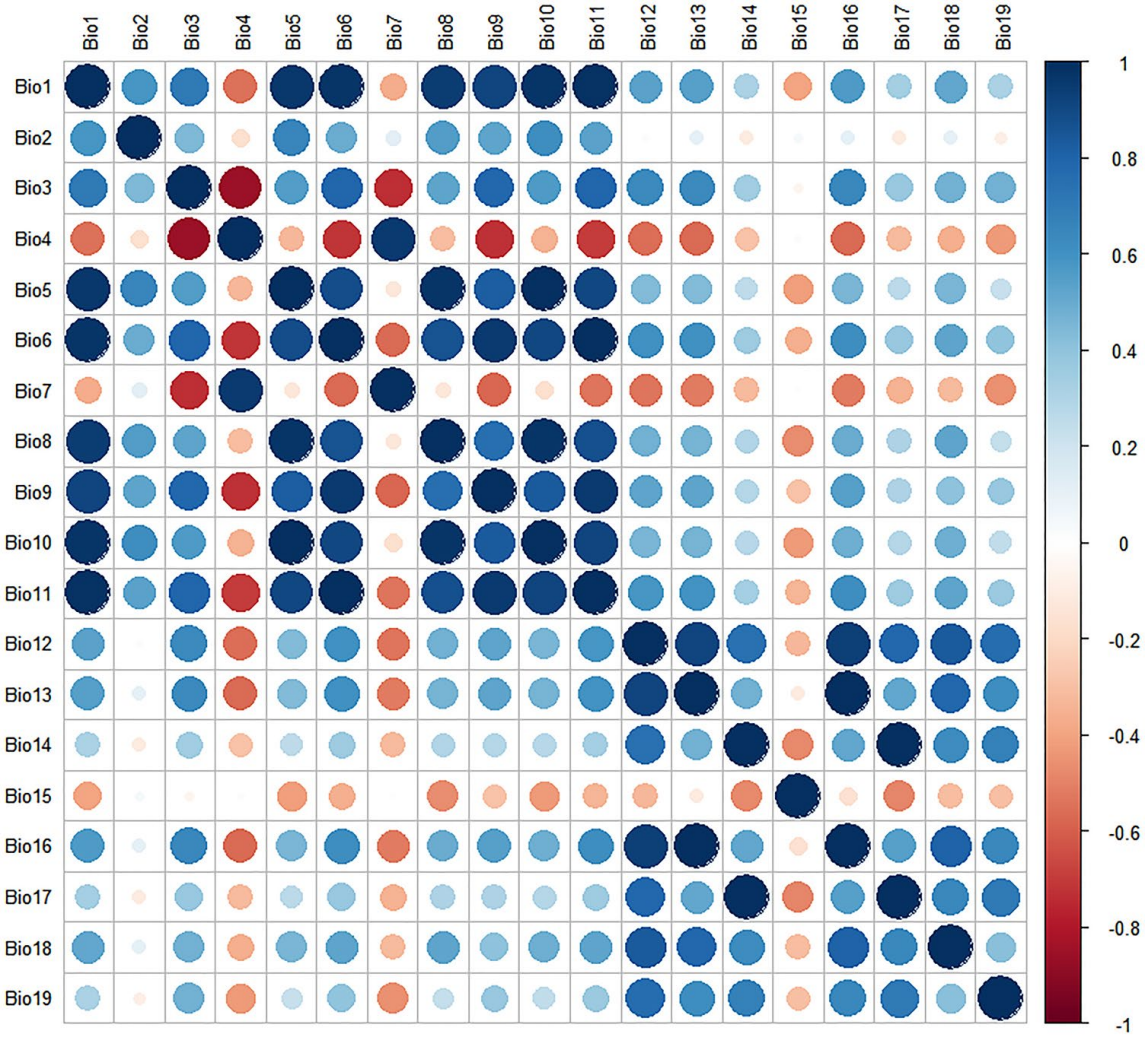
Correlation analysis of various environment variables. Created in R 4.1.2 (https://www.r-project.org/).

**Figure 10:**
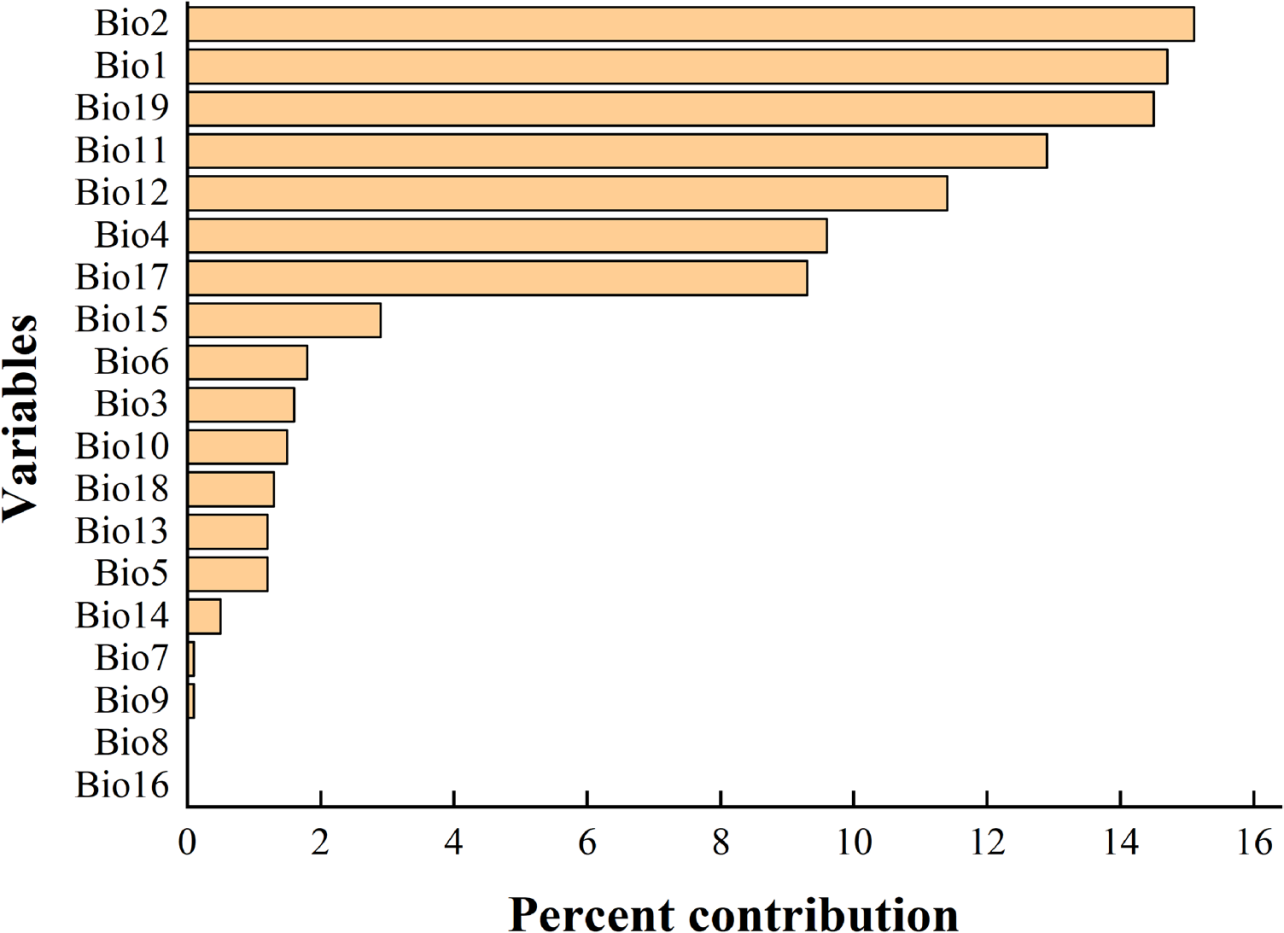
Percent contribution of 19 environmental variables to *N. californicus*.

Historical and future climate data were downloaded from the WorldClim website (https://www.worldclim.org/) and included 19 bioclimatic variables (2.5′ resolution) (Table 5). The historical climate data were the average values from 1970 to 2000. The future climate data (2030s, 2050s, 2070s and 2090s) were from three different scenarios SSP126, SSP245 and SSP585 under BCC-CSM2-MR mode. Second, the correlation analysis of 19 environmental variables (historical climate data) was carried out by using ENMtools software and use R package "corrplot"^67^ to draw heat map, and the contribution rate analysis was carried out by using MaxEnt software to import species data and environmental data. Environmental variables were determined to be suitable based on Pearson’s coefficients higher than |0.8| (very significant correlation) (Fig. 9) and contribution rates (Fig. 10).

**Table 5:** List of enviromental variables.

Variables	Full name	Variables	Full name
Bio1	Annual mean temperature	Bio11	Mean temperature of coldest quarter
Bio2	Mean diurnal range	Bio12	Annual precipitation
Bio3	Isothermality	Bio13	Precipitation of wettest month
Bio4	Temperature seasonality	Bio14	Precipitation of driest month
Bio5	Max temperature of warmest month	Bio15	Precipitation seasonality
Bio6	Min temperature of coldest month	Bio16	Precipitation of wettest quarter
Bio7	Temperature annual range	Bio17	Precipitation of driest quarter
Bio8	Mean temperature of wettest quarter	Bio18	Precipitation of warmest quarter
Bio9	Mean temperature of driest quarter	Bio19	Precipitation of coldest quarter
Bio10	Mean temperature of warmest quarter

After completing the above steps, six environmental variables were finally retained and used to build the final model, including annual mean temperature (Bio1), mean diurnal range (Bio2), temperature seasonality (Bio4), annual precipitation (Bio12), precipitation of driest quarter (Bio17), and precipitation of coldest quarter (Bio19).

### Model optimization

The ENMeval package^68^ in R was used to test the Akaike information criterion correction (AICc), which generally provides priority to parameters with small AICc values for simulation and is considered to be a standard measure of the goodness of fit of a model. Finally, we selected the model with a delta AICc equal to 0 according to the result of the ENMeval procedure.

### Distribution modelling with MaxEnt

The occurrence data and seven environmental variables of *N. californicus* were input into MaxEnt, and then ‘Create Response Curve’, ‘Do Jackknife to Measure Variable Importance’, and output format as ‘Logistic’ were selected. In the settings, the ‘Random test percentage’ was set to 25 (75% distribution points were randomly selected as the training set to build the model, and the remaining 25% distribution points were selected as the test set), and the ‘Regularization Multiplier’ was set to 1, ‘Replicates’ was set to 10 times; the model was set to ‘Hinge Features’ and the other parameters were set to the default software parameters. The receiver operating characteristic (ROC) curve output by MaxEnt was used to evaluate the accuracy of the model. Model performance was classified as failing (0.5**–**0.6), poor (0.6**–**0.7), fair (0.7**–**0.8), good (0.8**–**0.9), and excellent (0.9**-**) according to the AUC value^69^. Moreover, the True Skill Statistic (TSS) also have performed to test the performance of MaxEnt model^70^. For future potential distribution analysis of different scenarios, the corresponding future environment data will be placed in the ‘Projection Layers Directory/file’ in MaxEnt and run under the same settings.

### Distribution modelling

We imported the ASC II file obtained after the MaxEnt operation into ArcGIS 10.8.1, converted it into ‘raster’ format by using ‘ArcToolbox’, used the ‘Reclass’ function of the ‘Spatial Analyst tool’ to reclassify layers and divided regions according to their suitability level. Jenks' natural breaks were used to reclassify the suitability and classify the suitability into four categories: unsuitable area (P < 0.078008), lowly suitable area (0.078008 ≤ P < 0.269478), moderately suitable area (0.269478 ≤ P < 0.47513) and highly suitable area. Finally, based on a map of China, the potential distribution areas of *N. californicus* in China were extracted.

## Data Availability

Data from the current study are available from the corresponding author upon reasonable request.

## Code availability

R script is available from the corresponding author.
